# Infrastructuring Educational Genomics: Associations, Architectures, and Apparatuses

**DOI:** 10.1007/s42438-023-00451-3

**Published:** 2024-02-03

**Authors:** Ben Williamson, Dimitra Kotouza, Martyn Pickersgill, Jessica Pykett

**Affiliations:** 1 University of Edinburgh, Edinburgh, UK; 2 University of Birmingham, Birmingham, UK

**Keywords:** Behavior genetics, Bioinformatics, Educational genomics, Knowledge infrastructure, Sociogenomics

## Abstract

Technoscientific transformations in molecular genomics have begun to influence knowledge production in education. Interdisciplinary scientific consortia are seeking to identify ‘genetic influences’ on ‘educationally relevant’ traits, behaviors, and outcomes. This article examines the emerging ‘knowledge infrastructure’ of educational genomics, attending to the assembly and choreography of organizational associations, epistemic architecture, and technoscientific apparatuses implicated in the generation of genomic understandings from masses of bioinformation. As an infrastructure of datafied knowledge production, educational genomics is embedded in data-centered epistemologies and practices which recast educational problems in terms of molecular genetic associations—insights about which are deemed discoverable from digital bioinformation and potentially open to genetically informed interventions in policy and practice. While scientists claim to be ‘opening the black box of the genome’ and its association with educational outcomes, we open the black box of educational genomics itself as a source of emerging scientific authority. Data-intensive educational genomics does not straightforwardly ‘discover’ the biological bases of educationally relevant behaviors and outcomes. Rather, this knowledge infrastructure is also an experimental ‘ontological infrastructure’ supporting particular ways of knowing, understanding, explaining, and intervening in education, and recasting the human subjects of education as being surveyable and predictable through the algorithmic processing of bioinformation.

## Introduction

The genetic sciences have evolved historically alongside advances in data technologies. From the nineteenth-century origins of genetics as a statistical data practice (Porter 2018), it was later reconfigured in the mid-twentieth century through developments in information theory, cybernetics, and computer sciences, which reconceptualized life as ‘codes,’ ‘programming,’ and ‘information’ (Koopman 2020). As ‘big data’ became available for the computational formats of molecular research that emerged in the 2000s, genetics became a ‘genomic’ science utilizing computational systems and databases to interrogate DNA (Cambrosio et al. 2014). Since the human genome was sequenced, scientists in molecular genomics have utilized biostatistics methods and bioinformatics software to reconfigure understandings about the molecular structures and functions of living human bodies (Stevens 2013). The convergence of scientific practices with computational instruments in complex technoscientific infrastructures has made collecting and analyzing genomic data feasible, reshaping how research problems are identified and addressed, and rendering certain genetic conditions visible and knowable (Chow-White and García-Sancho 2012).

Genomic infrastructures are also being applied to psychological, behavioral, and social scientific research problems and public policy domains. Claims connecting genes to social, economic, and behavioral outcomes circulate widely within and beyond the fields of behavior genetics and ‘sociogenomics’ (Panofsky 2014; Bliss 2018). Social and behavior genomics studies of the purported genetic substrates of behaviors and social outcomes, and gene-environment interactions (Koellinger and Harden 2018), have been described by investigators as highly dependent upon the ‘rapid computational and technological progress’ made during the ‘big data revolution’ (Mills and Tropf 2020: 557). On this technological basis, scientists in social and behavior genomics aim ‘to open the black box of heritability’ using advanced computational methods (Harden and Koellinger 2020: 569), or ‘finally open the black box of the genome’ and ‘delve into the biological mechanisms and come up with a better understanding of the pathways from cells to society’ (Conley and Fletcher 2017: 35).

A principal public policy area targeted by social and behavior genomics is education. Social and behavior genomics scientists study what are framed as ‘genetic influences’ on educational outcomes, as signified by the interdisciplinary synthesis referred to by some as ‘educational genomics’ (Kovas et al. 2016) and characterized as a ‘genomic revolution for education research and policy’ (Morris et al. 2022: 1). By surveying genetic data, educational genomics attempts to predict how a person’s genotype—their unique, complete set of genetic material—influences a phenotype, or traits, behaviors, and outcomes that are considered to be educationally relevant (Thomas et al. 2015). Genomic analysis has been applied to the study of phenotypes including educational attainment, cognitive ability, school achievement, and various other traits, abilities, and behaviors (Sabatello 2018; Visscher 2022). Genomics is also promoted in educational policy discussions, particularly in the UK (Asbury et al. 2022; Government Office for Science 2022).

The application of genomics in research on educational matters is also the subject of significant scientific and bioethical controversy. There are ongoing contests over, for instance, the scientific practicality of distinguishing genetic influences from environmental influences (Burt 2023a), and the risks that using genetic information may lead to forms of biological reductionism, discrimination, stigmatization, and racism, as well as distracting from other forms of intervention (Parens and Meyer 2023). Education genomics research has thus been criticized both on scientific grounds for lacking ‘biologically realistic’ explanatory power and on socio-political lines for privileging somatic explanations over social structural analyses (Burt 2023b: 63).

While educational genomics aims to ‘open the black box’ of complex genetic associations with education, the aim of our analysis is to open the black box of educational genomics itself. We seek to better understand the scientific infrastructure—the organizational associations, epistemic architecture, and technoscientific apparatuses—that supports this field and its imaginaries, contributing to recent research examining the critical issues arising from syntheses of biology and education (e.g., Panofsky 2015; Gillborn 2016; Gulson and Baker 2018; Martschenko, Trejo, and Domingue 2019; Youdell and Lindley 2018; Pickersgill 2020; Martschenko 2021), especially their convergence with computational analysis (Gulson and Webb 2018; Williamson 2021; Means et al. 2022). Educational genomics research, and the discourse surrounding it, has potential to reshape how educational outcomes and problems are investigated, understood, and explained in terms of genetic influences and propensities, as well as to influence policy and practice interventions.

Our central argument is that data-intensive educational genomics do not straightforwardly ‘discover’ the biological bases of educationally relevant behaviors, traits, and phenotypes from data-mining bioinformation, or produce ‘biologically realistic’ or causal explanations of the genetic influences on educational outcomes, as its proponents suggest (Harden and Koellinger 2020). Rather, the scientific infrastructure being constructed and operationalized to enable and support educational genomics is also an experimental ‘ontological infrastructure’ (Jensen and Morita 2017). The distinctive ontology of educational genomics, in which genetic data processed in computers are taken as biologically realistic representations of biological matter, is therefore ‘the *consequence* of infrastructural arrangements’ (Jensen and Morita 2017: 618–619) (emphasis original). The ‘gene-centric worldview’ characterizing many efforts to identify ‘genetic influence’ in social and behavioral genomics research (Burt 2023b: 60) privileges biologically realistic explanations for highly complex, context-dependent social influences and outcomes. We reverse engineer this ontology by foregrounding the role of infrastructure in mediating and shaping the scientific knowledge produced by educational genomics research.

Crucially, we argue, educational genomics proceeds from a correlational ontology grounded in algorithmic associations (Kotliar and Grosglik 2023), rather than causal explanations of biological mechanisms (Matthews and Turkheimer 2022). The experimental infrastructure of educational genomics supports a ‘data-centric’ mode of research (Leonelli 2016) and shapes ways of knowing, understanding, explaining, and intervening in education. In the genetic sciences, computational and informational conceptions of human life have become characteristic of an ‘information-centric epistemology’ that links genetic codes to computer codes and treats ‘physiochemical reality’ as ‘information transfers’ (Koopman 2020: 9). Genetic sciences ultimately construct a human who can be known ‘from the statistical residue of [their] genome’ as a calculable and ‘statistical body’ (Stevens 2013: 221–222; van Baren-Nawrocka et al. 2020).

In contrast to claims that educational genomics can ‘discover’ a ‘realistic’ biological explanation of the genetic pathways from somatic substance to school outcomes, educational genomics instead calculates statistical correlations as part of its ‘ontological experimentation’ (Jensen and Morita 2017: 620) related to the human subjects of education. Therefore, just as genomics has begun to define and ‘format’ human bodies ‘as subjects of genetic data,’ and therefore to enable interventions that are based on genetic data and informational understandings of biology (Koopman 2020: 10), then educational genomics has begun formatting bioinformational subjects that may be identified and intervened upon from their rendering as genetic data. Such genetic formatting constitutes ontological experimentation in the analysis and definition of the biological correlates of educationally relevant behaviors and outcomes in educational genomics.

In other words, educational genomics formats a bioinformational substitute of the human subject as the potential basis for interventions in education policy and practice: a human subject whose genome may be data-mined, making educational outcomes legible and predictable from statistical correlations in bioinformation, and whose educational trajectory may then be shaped by genetically informed interventions. This surveyable and predictable bioinformational proxy is the product of ongoing efforts to construct and activate a scientific infrastructure of educational genomics. In the following analysis, we elaborate on how the construction of an infrastructure for educational genomics research enables these forms of ontological experimentation. First, though, we situate our analysis in conceptual research on scientific knowledge infrastructures, outline our methodological approach, and briefly summarize the historical precursors of contemporary educational genomics.

## Infrastructuring Genomics

Like other scientific domains of investigation and knowledge production, educational genomics is constituted through an accumulating sociotechnical ‘knowledge infrastructure’ consisting of people and organizations, epistemologies and practices, and technologies and methods (Edwards et al. 2013). As the following analysis demonstrates, the scientific knowledge infrastructure of educational genomics is currently taking shape through an imbrication of *associations*, *architectures*, and *apparatuses*. First, educational genomics is performed by large-scale, sometimes densely networked organizational and interpersonal *associations*, representing a consortia-driven ‘big biology’ mode of knowledge production. Second, educational genomics deploys a specific epistemic *architecture* for understanding biological influences on educationally relevant outcomes and behaviors. Third, methodological *apparatuses*, which heavily emphasize bioinformatic data mining and algorithmic discovery methods, are mobilized for data-intensive knowledge production.

These interacting and reciprocal associations, architectures, and apparatuses constitute educational genomics as a ‘science-in-the-making’ in which scientific instruments ‘actively mediate how reality becomes present to—and is treated by—scientists’ (de Boer, te Molder, and Verbeek 2021: 392). As such, educational genomics is not a settled or autonomous field of investigation, but a site of ongoing ‘infrastructuring’ that involves a complex accumulation of social, scientific, technical, material, political, and economic relations into a stabilized infrastructure of knowledge production (Blok et al. 2016: 5). ‘Infrastructuring’ highlights that ways of acting towards, knowing about, and valuing the objects of scientific investigation are inextricably bound up with the social, technical, and organizational practices and scientific instruments of large-scale computer-enabled information systems, which have to be designed, made, implemented, and maintained in order to allow scientific knowledge to be produced (Meckin 2020).

Transnational knowledge infrastructures and their instruments also embed particular assumptions and politics, which impact engagements with them and their epistemic and ontological affordances. Scientific ‘infrastructures include and exclude, and they enable certain kinds of knowledge and action rather than others’ as well as enabling attempts at ‘exercising control’ over the objects and subjects they are built to analyze (Blok et al. 2016: 6). Scientific knowledge infrastructures therefore function as ‘experimental systems’ consisting of interrelated devices, forms of practice and organization, and conceptual frames that facilitate the making of scientific knowledge, thus constituting ‘ontological experiments’ in how objects and subjects of investigation are conceived, interpreted, and explained (Jensen and Morita 2017).

Studies of data-centric biology have thus attended to how genomic knowledge production is interdependent with complex infrastructures of computational technologies and methodologies, which are reconfiguring how human biology is understood in terms of statistical correlations and patterns in large datasets (Chow-White and García-Sancho 2012; Stevens 2013; Leonelli 2016). Genomics ‘is ultimately a statistical exercise that depends on the analytic software itself and the information that goes into the statistical software’ (Fujimura and Rajagopalan 2011: 15). It consists of specific scientific sites and experts, epistemologies and practices, and methods and technologies of knowledge production and circulation, which generate datafied objects of attention that can move within and beyond research settings (Cruz 2022). We likewise approach educational genomics as a sociotechnical site of ongoing infrastructuring, paying careful attention to the associations, architectures, and apparatuses constitutive of this emerging domain of knowledge production and its claims to potential policy relevance.

For the analysis of associations, we identified core clusters of individual actors, institutions, and their interconnections, by conducting detailed web searches surfacing relevant details about the organizations and actors and their associations with research centers, projects, collaborators, conferences, and funders.^1^ Informed by the mapping of associations, we compiled an archive of more than 100 texts relevant to educational genomics, including research articles, books, reports, conference papers, and media articles published in English between 2005 and 2023.^2^ Concentrating particularly on a sample of texts produced by key nodal actors from the analysis of associations, observations were made of the repeated approaches, ideas, and claims common to the discourse of educational genomics, conceiving these as a conceptual architecture underpinning such research.

To examine the technological apparatuses, we produced characterizations of key technologies used in educational genomics, entailing close attention to the methodological appendices of published papers, and the following-up of references to bioinformatics applications. In concert with the conceptual architecture of bioscientists, bioinformatics apparatuses may have ontological effects, structuring particular ways of understanding human bodies, behaviors, or outcomes (Chow-White and García-Sancho 2012; de Boer et al. 2021). We further analyzed the discourses that frame such technologies; for example, what one prominent behavior geneticist refers to as their ‘visionary big-science’ capacity to ‘discover’ genetic differences and ‘transform’ scientific understanding of human behaviors (Plomin 2018: 123). Seven subsequent interviews with scientists involved in educational genomics consolidated our understanding of its infrastructural composition and implications. We now briefly survey the recent history of genetics and genomics in education, before moving to a substantive analysis of the associations, architectures, and apparatuses comprising educational genomics.

## Genetics and Genomics in Education

Genetics has a long history in education that has anticipated the recent emergence of educational genomics. Even before genetics was named as a field of inquiry, schools for so-called feeble-minded children collected numerical data to inform early studies of heredity, prefacing the late nineteenth-century co-development of statistics and eugenics (Porter 2018). The eugenics movement exerted significant influence on education during the twentieth century in the UK and the USA (Mazumdar 1992; Chitty 2007), especially through the use of intelligence quotient (IQ) testing and mathematical techniques to differentiate individuals according to genetically determinist claims about cognitive ability (Lowe 1980, 1998). Such intelligence tests constituted early psychometric attempts to ‘format’ students in terms of the biological correlates or causes of measurable mental aptitudes (Koopman 2020). In the postwar years, behavior genetics established itself as a research field dedicated to examining the impact of genetics on behaviors, despite ongoing controversies over its eugenic legacy (Panofsky 2014). Subsequently, from the 1960s onwards, behavior genetics produced a large body of research on the heritability of purportedly educationally relevant traits, including intelligence, attention, and other cognitive and noncognitive abilities, largely from methodological innovations in quantitative genetics (Plomin et al. 2007).

By the late 1980s, genetic explanations were becoming ‘particularly appealing in school systems pressed by demands for efficiency and accountability,’ leading to advocacy for strategies of genetic screening, prediction, and preventive intervention (Nelkin and Tancredi 1991: 51). Strategies of genetic screening and preventive intervention were advocated in relation to so-called problems arising within the classroom, with some predicting genetic tests could become ‘part of the standard testing regime in schools,’ as ‘objective assessments that have predictive value’ for ‘tailored regimes of learning’ (Rose 2007: 119–120). Inspired by emerging genomic understandings of ‘genetic polymorphism’ derived from molecular methods in the 1990s and 2000s, proposals circulated for a ‘genomics-education merger’ and new roles for ‘educogeneticists’ in schools, emphasizing interventions based on the ‘pattern of genetic variants’ in children’s genomes, which ‘should lead to improved individual learning outcomes and the maximization of the learning potential for every child’ (Grigorenko 2007: 24). As such, the early 2000s saw the foundations being laid for an infrastructure of educational genomics research that, animated by advances in molecular genomics, would proceed from the direct analysis of genetic data (Kovas et al. 2016).

Technoscientific developments in genomic methodologies have been promoted as a ‘genomic revolution’ for educational research and policy since around 2010 (Morris et al. 2022). Using data-intensive instruments and methods, behavior geneticists have begun studying what are taken to be traits and outcomes relevant to education (Malanchini et al. 2020), including cognitive ability, intelligence, educational attainment, achievement, and noncognitive skills (Selzam et al. 2017; Rimfeld et al. 2018; Demange et al. 2021). Sociogenomics research, which combines genomics and quantitative social sciences, and genoeconomics, the application of genomics in economics (Benjamin et al. 2012; Freese 2018; Braudt 2018; Mills and Tropf 2020), have extended genomic data analysis to a growing range of socio-economic outcomes and public policy areas like education (Domingue et al. 2015; Belsky et al. 2016; Cesarini and Visscher 2017). For some, contemporary genomic methods appear to make it possible to ‘personalize’ education around the individual’s genome, in a model termed ‘precision education’ and modelled after the biomedical approach of ‘precision medicine’ (Shakeshaft et al. 2013; Plomin 2018; Sokolowski and Ansari 2018; Shero et al. 2021).

The prospect of embryo selection and genetic editing based on DNA testing of educational potential has even become an area of bioethical debate in social and behavioral genomics (Meyer et al. 2023), signalling how ‘biodigital’ augmentations produced through genetic editing technologies have become a site of speculation in relation to education (Gulson and Webb 2018; Reader 2022). During this period, then, behavior genetics, sociogenomics, and genoeconomics have developed a major research agenda on genetics and education. This entails distinctive ways of conceiving the biological correlates of educational outcomes and has become possible due to the construction of a technoscientific infrastructure for data collection, analysis, and knowledge production.

The research enabled by the emerging infrastructure of educational genomics, the discourse supporting it, and claims of the relevance of its findings for education, has animated growing policy interest. This is notable in, for instance, the UK. Policy-facing agencies and scientific societies have begun circulating educational genomics evidence and proposals, particularly for genetic screening in the early years (Asbury et al. 2022). The UK Government Office for Science (2022: 136), for example, synthesized social and behavioral genetics research findings on education, highlighting how scientists have capitalized on medical genomics infrastructures to produce ‘insight into the biological architecture of learning and education processes,’ and suggesting its potential to ‘inform more beneficial interventions to improve pupils’ educational outcomes.’

Critics, however, argue that existing datasets and associated results are not representative across different populations (Herd et al. 2021), reinforce racialized categorizations and discriminatory outcomes (Roberts and Rollins 2020), produce negative self-fulfilling prophecies (Matthews et al. 2021), and are easily appropriated to support regressive and racist political agendas (Martschenko et al. 2019). Methodological controversies over measurement, prediction, and identification of genotype-phenotype mechanisms persist in social and behavior genomics too (Matthews and Turkheimer 2022), while ideas about ‘precision education’ are contested on practical, ethical, and scientific grounds (Sabatello et al. 2021). Indeed, these methodological and bioethical controversies are sources of ongoing debate and innovation within social and behavior genomics in both the UK and USA (Morris et al. 2022; Burt 2023a; Parens and Meyer 2023), highlighting how educational genomics remains enmeshed in a contested terrain of research while simultaneously extending its claims to biological authority on educational matters, as we now examine.

## Associations

In this first step of our analysis, we conceptualize the infrastructuring of educational genomics as a process of ‘harmonization’ (Ackerman et al. 2016), in which scientific institutions and individuals are interconnected by and cohere around genetic databases. Educational genomics depends on assembling relations between various research centers, institutions, associations, project teams, and individual scientists, as well as epistemic frames and sociotechnical apparatuses. As introductory remarks to a 2018 workshop entitled ‘Genes, Schools, and Interventions That Address Educational Inequality’ put it, the participants represented ‘overlapping Venn diagrams of social networks.’^3^ Researchers across social and behavior genomics have backgrounds in psychology, economics, sociology, political science, demography, epidemiology, behavior genetics, and quantitative genetics, as well as computer science, data science, biostatistics, and bioinformatics (Conley and Fletcher 2017). Consistent with the ‘big data,’ ‘big team,’ and ‘big funding’ character of genomics, social and behavior genomics is an interdisciplinary, multisector, and network-based scientific endeavor (Mills and Rahal 2019). Moreover, as in genomics, educational genomics is organized around large bioinformational databases, which constitute and sustain associations between researchers, teams, and epistemic programs (Chow-White and García-Sancho 2012), and reconfigure knowledge production in relation to educational matters as a ‘big biology’ enterprise (Vermeulen 2016).

Infrastructuring educational genomics therefore requires the harmonization of various disciplines and laboratory settings around the capacities of bioinformational databases. The work performed by these harmonizing agencies to infrastructure educational genomics is consequential to the kinds of research that can be performed, and thus to the forms of knowledge produced and the bioinformational configuration of the human subjects of education such studies entail. The Social Science Genetic Association Consortium (SSGAC) is the most established center in social genomics research related to education and plays a significant role as an infrastructural harmonizer and facilitator of such studies by bringing together multidisciplinary expertise, aggregating and making available data for analysis, developing computational methods, and solving logistical and sociolegal problems with data storage and security. As a ‘research infrastructure’ and a ‘multi-institutional, international research group,’ SSGAC operates as a distributed network across institutions and disciplines in a variety of principally high-income countries, most notably Australia, the Netherlands, and the USA, with team members also associated with organizations including the RAND Corporation and the National Bureau of Economic Research.^4^

SSGAC was founded with US National Science Foundation funding in 2011 by behavioral economists and genoeconomists as a research consortium to develop a large-data approach to social science genetics (Beauchamp et al. 2011). Further funding for its activities has been granted by major national science funding agencies and philanthropic foundations, including the US National Institute of Health, European Research Council, Swedish Research Council, Russell Sage Foundation, Open Philanthropy Project, and the Pershing Square Fund of the Foundations of Human Behavior. SSGAC specializes in meta-studies involving very large samples of data on phenotypes/traits, including attitudes, behaviors, economic preferences, and socioeconomic outcomes, as well as compiling, harmonizing, and sharing datasets publicly for re-use by others (Benjamin et al. 2012). Such ‘harmonization’ of both datasets and ‘harmonious scientists’ by multi-sited consortia is integral to accomplishing quantification standards and molecular precision in genomics (Ackerman et al. 2016: 194).

The consortium connects an even more distributed international network of researchers and centers that manage DNA banks associated with large genetic cohort studies.^5^ Data from more than 100 sources have been harmonized by SSGAC, with the largest provided by UK Biobank and the Silicon Valley personal genomics company 23andme (Becker et al. 2021). This has enabled SSGAC to produce four studies of educational attainment with escalating samples, most recently featuring more than 3 million genotyped individuals (Okbay et al. 2022). The materiality of these databases and the sociolegal and logistical processes that allow data sharing and co-analysis consequently have ontological effects in shaping the field in particular ways. Through generating and sharing DNA databases in such a coordinated and far-reaching fashion, educational genomics would not—in its current form—be imaginable and operable without the harmonizing research infrastructure of the SSGAC.

In this sense, SSGAC performs the significant function of not only harmonizing data, but also formatting it in ways that make it amenable to further analysis by other scientists. Its huge sample studies also provide benchmarks for how educational genomics studies can and should be performed, have become highly cited reference points that animate a wide range of follow-up studies, and provide evidentiary support to discourses promoting the potential of genetic analysis in educational practice and policy.

One well-known collaborator with SSGAC members is KP Harden of the Developmental Behavior Genetics lab, University of Texas, Austin. Harden is a highly cited behavior geneticist whose recent research primarily uses sociogenomics methods, with funding from the Templeton Foundation, Jacobs Foundation, and the US National Institute of Child Health and Human Development. She is closely associated with a range of other actors and institutions relating to educational genomics. In part, her traction relates to the wide publicity received for her popular science book *The Genetic Lottery* (Harden 2021). This, in turn, has generated wider interest in educational genomics, cultivated through explicitly ‘progressive’ opinion pieces and media interviews aimed at wider public and policy audiences (Lewis-Krause 2021). Indeed, by advocating what has been called a ‘new synthesis’ of social and genetic sciences (Jopling 2023), by synthesizing study findings by the SSGAC and others into accessible form, and by seeking to highlight how genetic information can be used to progressive ends of addressing social inequalities, *The Genetic Lottery* is itself a significant infrastructural connector. It has animated public, media, and political interest in the potential of genetic discovery and genetically informed interventions in education.

Other significant research nodes linked to SSGAC and Harden are based in the Netherlands and the UK. At Vrije Universiteit Amsterdam, sociogenomics researchers from economics and biological psychology departments received over €1.8 million funding from the European Research Council for the project ‘The molecular genetic architecture of educational attainment and its significance for cognitive health’ (Koellinger and Harden 2018). Various UK teams conduct behavioral and social genomics research. Most closely focused on education is the behavior genetics team at the Social Genetic and Developmental Psychology Centre, King’s College London, led by Robert Plomin. The center’s core funding is from the UK Medical Research Council, having received over £26 million since 1994, with other funders including the ERC, US National Institute of Health, British Academy, and Templeton Foundation.

The flagship Twins Early Development Study (TEDS) is based at the center and has likewise been running since 1994. TEDS data have been combined with SSGAC data to link educational attainment with cognitive measures of intelligence (Allegrini et al. 2019). Its methods encompass traditional twin studies as well as social genomics focusing predominantly on intelligence and cognitive development (Malanchini et al. 2020). Further, it is the institutional setting for ideas about genetically informed ‘precision education,’ as promoted in Plomin’s popular science book *Blueprint* (Plomin 2018), and proposals for ‘genetically sensitive schooling’ developed collaboratively with University of York academics studying behavioral genetics and education (Asbury and Plomin 2013; Plomin and von Stumm 2018). However, such proposals are largely contested by others working on educational genomics studies due to genetic data being deemed only weakly predictive and their potential to lead to deleterious outcomes (Parens and Meyer 2023). Nonetheless, these contested proposals serve to incite public and media interest and demonstrate how the harmonized data and findings of the SSGAC have been mobilized to support aims beyond the association’s own claims to agnosticism regarding the use of genetic information for policy or practice interventions.^6^

Other UK teams using social genomics methods are based at the universities of Bristol and Oxford. Researchers in the MRC Integrative Epidemiology Unit have investigated the genomics of education and economic outcomes with funding from the MRC, Economic and Social Research Council, Wellcome Trust, and Norwegian Research Council, and contribute datasets and advisory work to the SSGAC (Morris et al. 2018, 2020, 2022). The Leverhulme Centre on Demographic Science, funded with over £10 million by the Leverhulme Trust, develops sociogenomics methods and research (Mills and Tropf 2020), with relevant projects also funded by the ERC and the ESRC. Its lead researchers are also connected to the aforementioned teams in the USA, the Netherlands, and Australia. The Centre is a member of the European Social Science Genetics Network, along with seven other institutions, which established an EU-funded doctoral training network in 2022 to provide training ‘in state-of-the-art computational and bioinformatics methods for analyzing big data and in statistical techniques for empirical research’ for social genomics.^7^ Infrastructuring educational genomics is thus dependent on pedagogies of methodological training in bioinformatics and socialization of researchers into its knowledge community as well as investment in computing facilities and financial support through large-scale grant funding.

Besides formal institutional connections and networks, researchers applying genomics to education interact through a social infrastructure of conferences and workshops. These function as ‘community-making devices’ (Molyneux-Hodgson and Meyer 2009: 140), helping to propel epistemic developments and the promissory discourses that power these (Pickersgill 2023). Key conferences include the Integrating Genetics and the Social Sciences conference, hosted annually since 2010 at the University of Colorado, Boulder, the international Behavior Genetics Association annual conference, and the European Social Science Genetics Network annual conference established in 2022, as well as an annual SSGAC Summer Institute. Another is a series of conferences on genetics and social science at the University of Chicago Center for the Economics of Human Development in 2016, 2018, and 2021 with themes on polygenic prediction, genoeconomics, and educational interventions.

A series of workshops in the UK, coordinated on behalf of the Early Intervention Foundation (part of the UK government’s ‘What Works Network’) in 2020–2021, focused on generating policy advice on genetics for early years intervention, and included participation from the SSGAC, Plomin, Harden, and many others from both the UK and USA (Asbury, McBride, and Rimfield 2021). Its report was intended to generate and mobilize evidence and policy recommendations, and proposed the potential of ‘genetically informed social policy-making’ in the UK (Asbury et al. 2022: 8)—later leading to media coverage in the UK education press (Asbury 2023). A series of events hosted by the US bioethics research institute the Hastings Center, and co-directed by a bioethicist from the SSGAC, involved many social and behavioral genomics participants, leading to a ‘consensus report’ on the bioethical implications of such work (Parens and Meyer 2023).

Such reports and events not only support community-making among social and behavior genetics scientists, but function as translational techniques to turn complex scientific knowledge into publicly accessible artifacts that may circulate in media and policy spaces and potentially produce conviction in the idea that genetic data can be mobilized in social policy areas like education.

In sum, the application of genomics to education exemplifies a form of networked, consortia-driven science that advances through institutional and interpersonal associations across genomic, psychological, economic, social, and computer science disciplines and national borders, and which commonly converges around DNA databases (Chow-White and García-Sancho 2012). The associations that constitute educational genomics are characterized by processes of harmonization, consensus-building, translation, disciplinary synthesis, community-making, and the social and pedagogic practices that such connections entail. These harmonious relations materialize in large-scale ‘collective’ projects that resemble the composition of ‘big biology’ in the wider genomics field (Vermeulen 2016), characterized by disciplinary cross-fertilization of concepts and methods, large funding grants, publications with extensive authorship teams, and the creation and circulation of digital bioinformation via powerful computing infrastructure (Cambrosio et al. 2014).

As such, educational genomics would not be possible without the kind of harmonizing associations, networks, syntheses, and underpinning computing and data resources that characterize data-intensive genomics. Moreover, the associations responsible for constructing an infrastructure of educational genomics center digital bioinformation as a source for conceptualizing educational problems and proposing solutions. Harmonization of data is thus integral to the kinds of ontological experiments enacted by educational genomics research, and to its underpinning scientific epistemologies.

## Architectures

The networks and associations that constitute the infrastructure of educational genomics embed and enable a distinctive epistemic framework for conceptualizing the human subjects of education. The second step in our analysis is therefore to examine the epistemic architecture or conceptual framework of educational genomics, which we argue foregrounds a molecularized definition of educational outcomes and subjects that is possible only owing to the availability of high-powered computing instruments for processing genetic bioinformation.

Contemporary genomic sciences are characterized by a ‘molecular style of thought,’ which involves shared modes of ‘thinking, seeing, and practicing’ according to scientific consensus on the relevant objects of analysis, methods of inquiry, appropriate technical systems for data processing, and ways of identifying arguments and explanations (Rose 2007: 12). Such a molecular style of thought typifies educational genomics. Educational phenomena are conceptualized, investigated, and explained in terms of molecular genetic processes and interactions with other social and environmental factors (Koellinger and Harden 2018; Morris et al. 2022). Even where gene-environment interactions are highlighted, as in other domains of genomic science, phenomena are often ‘re-defined in terms of their molecular components,’ with research directed to ‘go into the body’ and ‘turn from efforts to understand social and environmental exposures outside the body, to quantifying their effects inside the body’ (Darling et al. 2016: 51).

This quantitative way of understanding and explaining the molecular basis of learning outcomes operates as an overarching epistemic architecture, or a conceptual and cognitive schema, that patterns and organizes knowledge production in educational genomics. The significance here is that a molecular epistemology, and the way it conceives and formats subjects, is inseparable from the computational instruments that format data and thereby make educational outcomes and behaviors visible, legible, and knowable from looking ‘into the body.’

This distinctive epistemic architecture derives from the so-called laws of behavior genetics infusing social and behavior genomics research, which state that all human behavior is heritable; environmental effects are smaller than the effect of genes; and substantial variation in behavioral traits is not accounted for by either genes or families (Turkheimer 2000). A ‘fourth law’ was proposed in 2015 by SSGAC and associated sociogenomics scientists ‘on the basis of molecular studies that have measured DNA variation directly,’ stating that ‘a typical human behavioral trait is associated with very many genetic variants, each of which accounts for a very small percentage of the behavioral variability’ (Chabris et al. 2015: 305). This attempt to create ongoing ‘laws’ grounded in emerging biological data contributes to a discourse of novelty and import associated with educational genomics, framing it as a field in evolution that demands ongoing definitional dialogue, while simultaneously asserting its authority as an epistemic architecture (Pickersgill 2021).

The fourth law was characterized on the basis of studies seeking patterns and associations among a multiplicity of genetic variants known as single nucleotide polymorphisms (SNPs). SNPs are tiny building blocks in human DNA, regarded as each having minuscule effects that function ‘additively’ through ‘polygenic’ associations to influence complex traits or outcomes; the search for and ‘discovery’ of these associations through genomic methods ostensibly makes it possible to produce a complete understanding of the underlying ‘genetic architecture’ of a phenotypic trait or outcome (Timpson et al. 2018). The search for SNPs is based on early 2000s conceptualizations of ‘genetic polymorphism’ whereby many genotypic variants were deemed to contribute to a phenotypic outcome or behavioral trait, and are critical objects of attention in behavior genetics, sociogenomics, and genoeconomics (Benjamin et al. 2012; Belsky et al. 2016). They form, for instance, a biological basis for educational genomics wherein ‘educational attainment’ is conceptualized as ‘a phenotype affected by thousands of undiscovered genetic variants, each responsible for a minuscule fraction of individual differences’ (Chabris et al. 2015: 305).

Accordingly, for educational genomics, the ‘appropriate response to the Fourth Law’ has been asserted to be ‘research strategies suited to the reality that most genetic effects on behavioral traits are very small,’ which are deemed to necessitate ‘much larger samples’ and new methods to identify polygenic associations (Chabris et al. 2015: 308). Through such claims-making, the ‘bigness’ of educational genomics sample sizes of the kind choreographed through the logistical infrastructure of SSGAC is justified and catalyzed. These variations have been identified in recent SSGAC research from studying sample sizes ‘from tens of thousands to millions,’ utilizing ‘growing statistical power to detect tiny effects on highly polygenic traits’ and the ‘associations of specific genetic markers with social scientific outcomes’ (Harden and Koellinger 2020: 569). The most recent SSGAC study of educational attainment included analysis of around 2.5 million SNPs, although its authors argue that ‘even larger samples will enable other analyses that have not yet been adequately powered’ (Okbay et al. 2022).

Genotyped SNP differences have thus become the basis of increasingly ‘high-powered’ educational genomics studies seeking the polygenic, molecular-level determinants of educationally relevant behavioral phenotypes and outcomes (Morris et al. 2022). Via the normative force of a legalistic framing structuring behavior genetics more broadly, educational genomics directs researchers to go into the body to discover SNPs and then combine them into models of the ‘genetic architecture’ that underpins educational achievement.

However, the expertise of educational genomics does not only *discover* polygenic SNP associations as molecular-architectural explanations for educational outcomes. It also actively *assembles* them as objects of analysis through the scientific infrastructure for measuring digitalized bioinformation. SNPs and polygenicity were historically conceptualized through technological and methodological developments in the late twentieth and early twenty-first centuries, the result of efforts by the biotechnology industry and genomics consortia to accelerate and automate DNA analysis (Kragh-Furbo et al. 2016; Rajagopalan and Fujimura 2018). The epistemic architecture educational genomics is, therefore, interdependent with methodological-technical innovations in bioinformation collection, storage, and analysis, with its knowledge claims actively mediated by large-scale bioinformatics apparatuses (de Boer et al. 2021).

These bioinformatics instruments function to format genetic data related to education: concretely, by enumerating the SNPs associated with educational outcomes, and then aggregating these SNPs into genetic architectures of educational phenomena such as attainment and achievement. They thus constitute powerful ways of defining educational outcomes and subjects by mining genetic bioinformation. Insofar as such studies ‘go into the body,’ they also delve into the bioinformatized proxy bodies that have been made surveyable and mineable in databases. Claims regarding the architectural polygenicity of educational outcomes are, then, not only an expression of a molecular style of thought associated with the extension of genomics knowledge into education, but artifacts of incorporating data science methods and genomic technologies into educational investigations. The molecular-biological epistemology of educational genomics, in other words, is also a data- or information-centric epistemology associated with big data technologies, as examined next.

## Apparatuses

While educational genomics depends on network associations and a conceptual architecture foregrounding molecular polygenicity, it also relies reciprocally on technoscientific instruments and apparatuses adopted and adapted from biomedical genomics. The third stage of our analysis consequently engages more fully with the instrumentation of educational genomics and the forms of experimentation and knowledge production it enables. Building the infrastructure of educational genomics to incorporate molecular technologies thus activates a particular configuration of a surveyable bioinformational subject as the potential basis for educational investigation and intervention.

In biomedical genomics, knowledge co-evolves with the invention of technical and methodological infrastructures of data collection, analysis, and communication (Chow-White and García-Sancho 2012). Genomics knowledge claims are, then, the products of particular convergences of technical and methodological instruments, along with distinctive modes of conceptualization that emerge in specific organizational and material situations (Leonelli 2016; de Boer et al. 2021). Central to genomics has been the development of bioinformatics, the synthesis of biological inquiry with computerized statistical techniques (Bartlett et al. 2017), resulting in ‘the reconfiguration of biology as a data-driven information science’ (Parry and Greenhough 2018: 6). Bioinformatics has enabled ways of aggregating and handling data that have reworked the settings, practices, and conceptual approaches of biological knowledge production (Cambrosio et al. 2014; Mackenzie 2003; Stevens 2013). Algorithmic instruments and apparatuses for data analysis, storage, sorting, searching, prediction, and more have therefore participated in the imagining and operations of genomics as a science dealing with exponentially increasing sample sizes and complexity of molecular associations (Reardon 2017).

Social and behavioral genomics scientists involved in education-focused research refer to the ‘powerful toolboxes’ of ‘statistical genetics’ as the methodological and technical underpinnings of their ‘genetically informed study designs’ (Harden and Koellinger 2020: 574). This includes proposals to genotype children and predict genetic influence on their outcomes. Such analyses require significant computing power ‘since genomic data are truly big data’: ‘computational demands are high and generally demand moving to a cluster computing environment and arranging considerable storage’ (Mills and Tropf 2020: 563). The specific algorithms and technologies selected and used by scientists shape how researchers perceive and understand their objects of study, ‘the kinds of questions and answers that genome biologists pose and attempt to answer’ (Stevens 2016: 353). Ultimately, such technologies and attendant techniques of investigation are enrolled into educational genomics to the extent that they make straightforward claims like this not only utterable but logical: ‘molecular genetic research, particularly recent cutting-edge advances in DNA-based methods, has furthered our knowledge and understanding of cognitive ability, academic performance and their association’ (Malanchini et al. 2020: 229–230). Three instruments adapted from biomedical genomics are especially significant in underpinning such claims in educational genomics: microarrays, biobanks, and polygenic data-mining software.

### Microarray Chips

The first instrument underpinning educational genomics is ‘DNA microarray chips’ (also known as ‘SNP chips’ or ‘labs-on-a-chip’). DNA microarray chips, originating in medical genetics in the mid-2000s, were created and are manufactured by global biotechnology companies to identify variation between genotyped individuals (Rajagopalan and Fujimura 2018), consistent with how commercial genotyping and bioinformatics companies have historically entered into and shaped genomic knowledge discovery (Stevens 2021). Microarrays are small glass slides imprinted with DNA fragments; when a dissolved sample of DNA flows across the slide, it binds with complementary fragments, generating an optical signal at certain wavelengths of light for measurement by a detection instrument. Microarray scanners, or automated ‘laboratory robots’ capable of analyzing thousands of arrays, can then genotype an individual in terms of how they differ from a group or population across very large samples (Kragh-Furbo et al. 2016). Behavior geneticists involved in education-focused studies claim microarrays represent a ‘breakthrough in DNA research,’ making it possible ‘to genotype inexpensively and quickly the most common type of inherited DNA difference’ (von Stumm et al. 2020: 3).

Genotyping microarray platforms used for educational genomics studies are part of complex bioeconomic arrangements, multisector partnerships, and subcontracting agreements that constitute the contemporary technoscientific field of genomics (Birch 2017). Methodological innovations in social and behavior genomics have been argued to be the result of ‘radical drops in the cost of genome sequencing and growth in computational power,’ which ‘precipitated an unprecedented explosion of data, novel methods, applications, and results’ (Mills and Tropf 2020: 554). The apparatuses of genotyping SNP chips and scanners, which include international biotechnology organizations like Illumina and 23andme, represent key sociotechnical elements of the infrastructure of knowledge production in educational genomics. Researchers have argued DNA chips can be used ‘to predict strengths and weakness for individual pupils’ (Asbury and Plomin 2013: 12). ‘Gene chips’ have been speculatively invoked as a source of ‘individual genetic information to help children in school settings,’ and of ‘anticipatory guidance, based on genetic information,’ which could ‘result in more effective pedagogical approaches and beneficial outcomes’ (Grigorenko 2007: 24). More recently, Plomin (2018: 134) referred to ‘Learning Chips’—from which polygenic predictions for educational achievement could be derived—as ‘DNA fortune tellers,’ actively proposing their use as a source of anticipatory guidance in education.

One paper, ‘Predicting educational achievement from genomic measures and socioeconomic status,’ involved microarrays to study swab samples collected from approximately 5000 UK school children (von Stumm et al. 2020). Another team leveraged genotypes from 3500 UK school children, seeking to investigate ‘how accurately polygenic scores for education predicted pupils’ test score achievement’ (Morris et al. 2020: 1). The methods section from the report of this work indicated the range of biotechnology organizations involved in providing ‘chip genotyping platforms’ for educational genomics studies and the significant interpolation of private enterprise within a publicly funded and, ostensibly, -orientated endeavor, and hence the imbrication of (educational) genomics within the broader bioeconomy:

DNA of the […] children was extracted from blood, cell line and mouthwash samples, then […] genotyped using the Illumina HumanHap550 quad chip genotyping platforms by 23andme subcontracting the Wellcome Trust Sanger Institute […] and the Laboratory Corporation of America. (Morris et al. 2020: 11)

Microarrays highlight how the study of human biology has become centered on masses of data and analytical algorithms, as commercially produced bioinformatics apparatuses have intervened in methods and knowledge-making practices (Keating and Cambrosio 2012). Moreover, the design, statistical power, and technical constraints of the chips have shaped the ‘definition and significance of human genetic differences,’ through having ‘locked in’ the primary ‘conceptual frameworks humans should use to consider, organize, work with, and ultimately act on genetic differences’ (Rajagopalan and Fujimura 2018: 862). Polygenic variation in educational outcomes has therefore been ‘locked-in’ to educational genomics by the design and constraints of DNA chips developed in large part by global biotechnology companies. Microarrays have enabled a particular style of molecular analysis—and hence imagining—of human difference, consolidating visions for a form of research into educationally relevant genetic variations and proposals to use genetic data for anticipatory interventions.

Significantly, microarrays are complex sociotechnical and bioeconomic assemblages for interpreting and in a sense ‘producing’ human genetic variation, based on the biomedical application of techniques from the computing and data sciences including data mining, machine learning, computational algorithms, robotics, and automation (Kragh-Furbo et al. 2016). Accordingly, microarrays represent how educational genomics is intertwined with technical innovations, specific material objects, biomedical methodological practices, and bioeconomic valuation in the biotechnology industry. They make human subjects surveyable as bioinformational proxies with phenotypic traits and outcomes that can be predicted from genotyped samples. These technoscientific ‘lock-ins’ are further consolidated as genotyped microarray data are aggregated into large-scale bioinformation repositories.

### Biobank Repositories

Educational genomics depends on building infrastructural connections to ‘biobanks’ for access to the necessary genotyped data. Biobanks are large-scale repositories of biomedical samples and associated information for use in research and medicine. As ‘biorepositories’ that blur the distinction between the ‘wet lab’ of human specimen analysis and ‘dry lab’ data analytics, biobanks are also ‘nodes’ for ‘global sharing’ that are embroiled within sometimes diverging economic, epistemic, and normative agendas (Argudo-Portal and Domènech 2020: 1). In essence, they allow for the storage and exchange of ‘bioinformatized’ data. Following Chow-White and García-Sancho’s (2012) analysis of the emergence of DNA databases in the 1960s, the contemporary use of biobanks in educational genomics can be understood as representing a material-technical instantiation of the historical and ongoing convergence of biological and computer sciences, and of academia and industry. They represent a notably powerful point at which genetic data are formatted.

Biobanks are key sources of genotyped SNP data used in educational genomics. The SSGAC has arranged an array of biobank and cohort study data to calculate polygenic scores for educational attainment. This was, first, with a sample of 126,500 genotyped individuals, which identified three SNPs associated with just 2% of the variation in educational attainment (Rietveld et al. 2013), then a follow-up 2016 sample of 300,000 identifying 74 SNPs regarded as explaining 3.2% of variation (Okbay et al. 2016). In 2018, with an updated sample of 1.1 million, SSGAC scientists reported more than 1200 SNPs accounting for 11–13% of the variation in years spent in school (Lee et al. 2018). The million-sample database it constructed was based primarily on combining data sourced from the UK Biobank, a large-scale biomedical database funded by UK research councils and charities, and data from the aforementioned 23andme (proprietor of one of the world’s largest private biobanks).

The SSGAC has also completed another study with a scaled-up sample of 3 million through its extended partnership with 23andme, identifying 3952 lead SNPs explaining between 12 and 16% of the variation in educational attainment (Okbay et al. 2022). It further published an updateable ‘repository’ detailing the polygenicity of 47 distinctive phenotypes (five of which are educationally relevant) based on data sources including 23andme and UK Biobank (Becker et al. 2021). These thousands of polygenic associations constitute the genetic architecture of educational attainment, according to the SSGAC and the wider domain of educational genomics it supports. In other words, large-scale biobank data, and associated practices of storing and sharing bioinformation, have made it possible to conceive of the genetic or ‘biological architecture’ of learning outcomes as a potential object of policy attention (Government Office for Science 2022).

Biobanks are, however, far from merely neutral repositories of bioinformation, despite their enrolment in epistemic programs ostensibly generative of insights about both populations and, increasingly, the individuals inscribed within them (Hoeyer et al. 2019). The construction of the SSGAC datasets from biobanks can be understood as the result of ‘bioprospecting’ for data that holds potential value for further research and its ‘data packaging’ for re-use. Bioprospecting and packaging involve the ‘selection, formatting, standardization, and classification, as well as the development of methods for retrieval, analysis, visualization and quality control’ that underpin the integration of biological data for dissemination and analysis (Leonelli 2016: 16). The SSGAC thus bioprospects data from biobanks, negotiates financial contracts and legal data-sharing agreements, and packages data into ‘harmonized’ large-scale repositories and polygenic indices for sharing and analysis by other researchers (Becker et al. 2021). The bioprospecting and packaging of harmonized biobank data is central to the potential research yield of educational genomics, constituting an integral part of its infrastructure of datafied knowledge production.

Importantly, however, biobank data are constrained and limited in several ways. They tend only to include white European ancestry populations, and are not representative of diverse groups, skewing the ontological claims emerging from their use and potentially leading to discriminatory outcomes (Lee 2015; Herd et al. 2021). Further, how they are curated and modified and who gets to access their data shape the science that involves them (Argudo-Portal and Domènech 2020; Milanovic et al. 2018). This contouring is again tightly linked to bioeconomic dynamics, with private biobanks like 23andme amassing significant value by acting as ‘platforms’ and ‘two-sided markets’ generating capital from both consumers who provide their data for a fee and from research labs who pay to access the repository (Stoeklé et al. 2016). Organizations such as the SSGAC have to negotiate legal and logistical contracts over access to biobank data, data sharing, and security. As such, educational genomics depends on the commercial infrastructures of biotech companies and the forms of bioeconomic value-generation they entail (Birch 2017).

For the SSGAC and associated scientists, educational genomics studies have become feasible because biobanks routinely collect basic educational attainment information too (Benjamin et al. 2012). The biobanks underpinning educational genomics, then, make the data for such studies ‘conveniently available’ (Burt 2013b: 60), while shaping, defining, and constraining the kinds of data available, the analyses that can be conducted with them, and the claims made based upon them about the polygenic genetic architecture of educational outcomes. The biological architecture of educational outcomes claimed by educational genomics is to a large extent a bioinformational artifact of the technical, financial, legal, and logistical arrangements that constitute biobanks. These infrastructural arrangements make genotypes surveyable for genetic patterns related to educational outcomes through data mining software and techniques.

### Polygenic Data Mining

Biobanks and microarrays have together enabled the conduct of genome-wide association studies (GWAS) and the construction of polygenic scores (PGS) related to educational outcomes. The creation of polygenic scores, we suggest, constitutes a significant formatting of genetic data into a single numerical signal of genetic influence on educational outcomes that, it is claimed, provides a probabilistic prediction of an individual’s future prospects (Visscher 2022). It is this formatting of genetic predictors that has animated promotional rhetoric in educational genomics research (Plomin 2018; Harden 2021), and captured policy attention (Asbury et al. 2021).

GWAS, a form of big data analysis that involves surveying genomic bioinformation to find SNP variants statistically associated with a specific phenotype (Uffelmann et al. 2021), have principally been used to ‘search for genetic markers that may increase the risk of developing common complex diseases’ (Fujimura and Rajagopalan 2011: 8). Increasingly, they are also enrolled to study phenotypical behavioral traits and their genotypical correlates. As Fujimura and Rajagopalan (2011: 8) note, ‘GWAS researchers scan the genomes of large groups of individuals in search of genetic markers […] through statistical analyses that distinguish differences in the frequencies of genetic marker variants in cases afflicted with a disease versus controls.’ GWAS is a prominent tool within the wider apparatuses of genomics, and integral to producing polygenic scores in educational genomics.

Historically, polygenic scores emerged as a ‘pragmatic solution’ to the statistical problem of processing a very large number of small SNP associations in GWAS research (Janssens 2019: 147). A polygenic score is a single aggregated number that summarizes all the genetic variations in an individual in relation to a phenotype (outcome, trait, or behavior) of interest and is the central focus of most educational genomics studies (Domingue et al. 2015). PGS are calculated using bioinformatics applications, computing formats, algorithms, and statistical standards created by statistical and bioinformatics specialists in genomics research laboratories (Choi et al. 2020).

Commonly used in educational genomics, for example, GCTA (Genome-wide Complex Trait Analysis) is a freely available, ‘user friendly’ software package combining multiple statistical algorithms to estimate ‘additive genetic variation that is captured by SNP arrays and is therefore informative with respect to the genetic architecture of complex traits’ (Yang et al. 2011: 80). Likewise, PLINK is a ‘user friendly’ open-source genetic analysis software toolset for ‘computationally efficient’ GWAS, which makes ‘large data sets comprising hundreds of thousands of markers genotyped for thousands of individuals’ available to be ‘rapidly manipulated and analyzed’ to identify ‘polygenic effects’ (Purcell et al. 2007: 559).

Other applications utilized in educational genomics are PRSice and LDpred for calculating polygenic scores. LDpred—used by the SSGAC in its education studies—is a popular and computationally intensive bioinformatics method for ‘deriving polygenic scores based on summary statistics and a matrix of correlation between genetic variants’ (Privé et al. 2020: 5424). Likewise, PRsice is ‘an efficient and scalable software program for automating and simplifying’ the ‘computationally intensive’ process of calculating polygenic scores from large-scale biobank data (Choi and O’Reilly 2019: 1). PRSice is characterized as being able to ‘infer the genetic architecture of a trait’ at ‘high resolution’ (Euesden, Lewis, and O’Reilly 2015: 1466). The polygenic associations underpinning claims about genetic influence on educational outcomes are therefore artifacts of the convergence of high-powered genomic data mining software with molecular conceptualizations of somatic substance.

Automated ‘bioannotation’ software also generates ‘explanatory notes based on what is known about genomic and cellular biology’ to add descriptive power to data about polygenic association and genetic architecture (Harden 2021: 137). Genome annotation applications such as BLAST are used in educational genomics studies by the SSGAC to automatically describe the mechanisms of selected SNPs associated with educational outcomes (Lee et al. 2018), though these mechanistic explanations remain highly partial (Matthews and Turkheimer 2022). The dependency of researchers on such tools demonstrates how knowledge about the genetic dimensions, associations, and architecture of educationally relevant outcomes is inseparable from computational innovations, algorithms, and automation that have been central to both genomics and the bioeconomy.

In educational genomics literature, GWAS and PGS are presented as transformative methodologies for predicting and explaining the genetic dimensions of educationally relevant traits and learning outcomes (Allegrini et al. 2019; Plomin and von Stumm 2021). As Harden (2021: 58) has remarked: ‘A GWAS measures millions of SNPs in thousands of people and correlates each SNP with a phenotype’ like ‘years of education.’ Researchers can then ‘add up all the information across all SNPs into a single number’—a PGS—which can be used to ‘predict’ educational attainment (Harden 2021: 65). In turn, PGS are described as part of a purportedly new ‘genetic toolbox’ for ‘doing better social science research’ and inform education policy and practice interventions (Harden 2021: 188). PGS are framed as representing ‘an unbiased but noisy measure of what we call the “additive SNP factor,” which is the best linear predictor of the phenotype from the measured genetic variants’ (Becker et al. 2021: 1745).

The word ‘unbiased’ is key here. The framing of GWAS, polygenic scoring, and bioannotation commonly highlight a form of supposedly ‘theory-free’ or ‘hypothesis-free’ discovery science (Leonelli 2016). The bioinformatic data mining methodologies of GWAS, it is claimed, adopt ‘an unbiased, hypothesis-free approach to discover SNPs that are associated with a trait’ (Mills and Tropf 2020: 556). GWAS are described as a ‘technological advance’ that ‘enabled an atheoretical approach to identify associations across the genome,’ which has led to ‘increasingly more insight into the molecular genetic architecture of cognitive ability and academic performance’ (Malanchini et al. 2020: 235). Similarly, microarray chips are deemed to have enabled ‘atheoretical’ and ‘hypothesis-free investigation’ of the genetic aspects of ‘important social outcomes’ like education (Conley and Fletcher 2017: 44). The implication is that such ‘objective,’ and ‘unbiased’ discovery might become the basis for genetically informed educational interventions of the kind popularized in recent educational genomics books like *Blueprint* (Plomin 2018) and *The Genetic Lottery* (Harden 2021).

Yet, claims that polygenic scores ‘work’ as measures of ‘genetic propensities’ or represent ‘genetic influence’ on educational outcomes are highly disputed as ‘obscuring environmental influences’ and ‘perpetuating a flawed concept of genetic potential for social behaviors and achievements’ (Burt 2023a: 2). While debates continue about using genomic technologies such as GWAS and PGS for revealing educationally relevant knowledge, the extent to which such techniques may function both as scientific instruments that ‘mediate’ and ‘produce’ certain understandings of scientific phenomena (de Boer et al. 2021) and as political artifacts is less considered (Fujimura and Rajagopalan 2011).

The building of the microarray chips, the selection of markers, the genotyping itself, the selection of biobank data, the design of the GWAS software, and the automated calculation of polygenic scores all shape the knowledge produced in educational genomics. These impose molecular understandings of social problems, privileging and hardening biological explanations while obscuring the social dimensions of educational outcomes. Accordingly, the bioinformatics apparatus underpinning educational genomics is also a political apparatus that locks in a particular molecular conceptualization and statistical configuration of the underlying genetic architecture of educational outcomes. The apparatus of microarrays, biobanks, and polygenic scoring software formats genetic data in ways that configure knowledge about genetic influences on educational outcomes, privileging not just biological but bioinformational explanations as the basis for potential educational interventions.

The production of PGS for education is therefore synthesized through an apparatus of bioinformatics hardware and software packages, each loaded with algorithmic calculating tools that ultimately enable and mediate the production of specific knowledge claims. GWAS and PGS together represent a computational epistemology that assumes the heritability of complex human behaviors and social outcomes can be explained in finer polygenic grain and with more predictive confidence by increasing the quantitative and computational powers of analysis (Stevens 2013). The genetic influences on educational outcomes are regarded as objectively discoverable by data mining millions of bioinformational data points. This conception is folded unto understandings of GWAS and PGS as a means to develop supposedly better, more predictive, and actionable knowledge about educationally relevant phenotypes and outcomes.

Ultimately, polygenic scores are the end result of a series of formatting operations. Bioinformation produced using microarrays, aggregated and stored in biobanks, and analyzed through data mining software, produce a single numerical predictor of an individual’s probably educational prospects. It is this formatted quantitative signal of genetic influence on educational outcomes—generated through the infrastructure of network associations, conceptual architecture, and technoscientific apparatus outlined in this analysis—that has begun to animate promissory discourses and catalyze significant public, media, and policy interest in the use of genetic data in education.

## Conclusion

In this article, we have documented and analyzed some key constitutive elements and reciprocal relations of an emerging knowledge infrastructure for educational genomics, at a time of growing advocacy for genetically informed educational research, practice, and policy. This advocacy for opening up the ‘black box of the genome’ with molecular methods in education is reflected in large grants from funders, media interest, and increasing policy attention. Claims that genetic data could be used for screening children in the early years, investigating biological differences between groups, or to ‘target educational interventions’ and create ‘personalized’ pedagogic approaches, have become the subject of UK policy–facing reports (Government Office for Science 2022: 134; Asbury et al. 2021). In this context, our aim was to open the ‘black box’ of the organizational associations, epistemic architecture, and methodological apparatuses that are converging to comprise a knowledge infrastructure of educational genomics.

As a science-in-the-making undergoing infrastructuring, educational genomics aims to advance ‘a more comprehensive, biologically oriented model of individual differences in cognitive ability and learning’ (Malanchini et al. 2020: 230), framing its aim as the identification of ‘authentic genetic signals’ and biological influences on educational outcomes (Burt 2023b: 61). The knowledge claims of educational genomics are possible only due to the construction and operations of an underpinning scientific knowledge infrastructure, which profoundly shapes how studies are conducted, supports assertions of scientific authority and policy relevance, and formats human subjects as bioinformational proxies that can be searched in databases using bioinformatics software. We offer three key points in conclusion.

First, understood as undergoing infrastructuring, educational genomics is ‘inextricably bound up with the technical, social, and organizational practices of large-scale computer-enabled information infrastructures’ (Blok et al. 2016: 7). This infrastructuring enables ‘ontological experiments’ (Jensen and Morita 2017), reconstituting educational outcomes in terms of the ‘genetic architecture’ of thousands of polygenic molecular associations. The knowledge-making systems of educational genomics act as an ontological infrastructure for purportedly objective forms of knowing and practicing in education. Its emphasis on the genetic architecture of educational achievement, made legible at the molecular scale via bioinformatics, locks in polygenic conceptualizations of the genetic influences of learning and configures bioinformational educational subjects as genetically surveyable and predictable. By ordering epistemic activities through the convergence of algorithmic techniques and a molecular style of thought, the knowledge infrastructure of educational genomics may privilege and harden bioinformational explanations for the complex social factors underpinning academic achievement. In so doing, the social and environmental factors that underpin social and educational inequalities can be treated as biological qualities that are discoverable in the body (Darling et al. 2016).

Second, this potential is in part a corollary of the purportedly ‘unbiased’ and ‘hypothesis-free’ apparatuses—biobanks, SNP chips, GWAS, and PGS—that underpin knowledge production in educational genomics. These data-centric approaches proceed from the computational search for correlational patterns and associations with automated data mining algorithms rather than explicitly theory-centered inquiry (Kotliar and Grosglik 2023). They are, however, far from atheoretical, but embedded in ‘networks of concepts’ and ‘ways of seeing the biological world that guide scientific reasoning and the direction of research’ (Leonelli 2019: 2). Rather than offering ‘biologically realistic’ discoveries and explanations of the genetic mechanisms underlying educational outcomes, educational genomics privileges algorithmic correlations over causal biological explanations, with data mining for polygenic signals configuring a different ‘biological reality’ through algorithmically sorted associations (Janssens 2019).

The data-centric or information-centric epistemology characteristic of contemporary genomics formats human subjects in terms of genetic data and informational conceptions of biology (Koopman 2020). As such, the bioinformational epistemology that infuses efforts at infrastructuring educational genomics also carries ontological import. Specifically, it configures what biological and genetic influences are taken to be, with those conceptualizations then reified through a cascade of microarrays, biobanks, and polygenic data mining apparatuses. This comes with the potential for disparate impacts, as particular genetic influences are proposed as the basis for interventions in policy and practice.

Third, the combination of genetics and computation also helps grant social and behavioral genomics a license to produce and circulate seemingly authoritative claims about education and related social outcomes. This is reflected in assertions it will transform social science and generate ‘a more realistic understanding of human behavior and the functioning of societies’ (Harden and Koellinger 2020: 567), making ideas about ‘precision education’ based on polygenic scores or ‘genetically informed’ education policy utterable. Educational genomics claims objective biological authority and diminishes other forms of explanation related to education. Some specialists in this area actively undermine so-called genome blind social sciences for their alleged ‘failure’ to inform value-for-money policy interventions or educational reforms (Harden 2021: 234).

As in other areas of scientific innovation, knowledge infrastructures associated with educational genomics ‘may disadvantage and devalue older forms of knowledge production’ (Edwards et al. 2013: 11), while rhetorical claims to novelty themselves act as part of the machinery through which the import of educational genomics is framed and its salience asserted (Pickersgill 2021, 2023). As such, educational genomics can undermine many forms of social scientific analysis while advancing existing modes of large-scale statistical research to privilege biological investigation in educational research.

Through its current infrastructuring, educational genomics represents an emerging source of power and authority offering avowedly ‘realistic’ biological explanations for complex, socially situated behaviors and outcomes, while devaluing other forms of knowledge production or claims to reformatory authority. It recasts social phenomena as phenotypes that are substantially decodable from digital genotype data through algorithmic apparatuses and makes the human subjects of education legible as statistically surveyable and predictable bioinformational proxies. While the application of genomics to education remains highly contested on scientific, political, and bioethical lines, it deserves continued critical attention as its scientific knowledge infrastructure solidifies, knowledge production intensifies, and genetically informed policy action is encouraged. This paper contributes to important emerging scholarship on the intersections of biology, technology, and education (Peters, Jandrić, and Hayes 2022), and points to the need for additional future research to engage with the knowledge claims of educational genomics and the translation of such findings into proposals for genetically informed interventions in education policy and practice.

## Footnotes

1 We used social network graphing methods to map the social relations and organizational associations that characterize educational genomics, and annotated the data using the graph database management system Neo4j, visualizing educational genomics as a single map of social and organizational relationships. We used the annotated social graph of educational genomics as the basis for producing descriptions of the key nodal organizations and individuals, and their interorganizational and interpersonal associations.

2 For the textual analysis, we searched electronic catalogues for published research articles, reports, and media articles which used, reviewed, or critically analyzed using genomics methods to explore educational issues, covering the period 2005–2023, and compiled these texts using the bibliographic software Zotero. In particular, we searched for publications produced by research groups and individual scholars identified through the network mapping. We manually checked references and citations from the most recent and highly cited sources to ensure inclusion of relevant previous studies. These searches produced a corpus of over 100 research articles, as well as media articles, four full book-length treatments, and three relevant policy reports. Web searches also surfaced conference paper proceedings and video recordings, podcasts, and media appearances, which supplemented the textual dataset.

3 See https://cehd.uchicago.edu/?page_id=1400. Accessed 18 December 2023.

4 See https://www.thessgac.org/. Accessed 18 December 2023.

5 See https://www.thessgac.org/participating-cohorts. Accessed 18 December 2023.

6 See https://www.thessgac.org/faqs. Accessed 18 December 2023.

7 See https://euraxess.ec.europa.eu/jobs/857404. Accessed 18 December 2023.

## Declarations

### Disclaimer

This manuscript is composed of original material that has not previously been published and is not being considered elsewhere.
